# Knowledge and Awareness of Mothers of Asthmatic Children and Its Impact on Asthma Control: A Cross-Sectional Study From Qassim Region, Saudi Arabia

**DOI:** 10.7759/cureus.62880

**Published:** 2024-06-21

**Authors:** Rand M Alsalamah, Amel Sulaiman

**Affiliations:** 1 Family Medicine, Family Medicine Academy, Qassim Health Cluster, Buraidah, SAU

**Keywords:** attitude, parental knowledge, children, management, risk factors, asthma

## Abstract

Introduction

Asthma, a common chronic airway disorder, presents challenges in diagnosis and management, particularly in children. Triggers include allergens and pollutants, necessitating lifestyle modifications and pharmacological treatments. Severe cases require tailored management. International guidelines provide stepwise approaches, while the Saudi Thoracic Society offers comprehensive recommendations, emphasizing gradual treatment phases and thorough clinical assessment. This study aimed to evaluate the knowledge and awareness levels among mothers of asthmatic children in Qassim region, Saudi Arabia.

Methodology

This cross-sectional study was conducted among 422 mothers with asthmatic children at primary healthcare centers in Qassim, Saudi Arabia. Mothers' knowledge of asthma was assessed using an online questionnaire. Participants were selected via a convenient non-probability sampling technique. Data was cleaned in Excel and analyzed using IBM SPSS Statistics for Windows, Version 29 (Released 2023; IBM Corp., Armonk, New York, United States). Participants were selected via a convenient non-probability sampling technique. Necessary statistical tests like Chi-square and Fisher's exact test were applied.

Results

Our study involved 422 mothers of asthmatic children in Saudi Arabia. Most participants were aged 35-44 years (50.7%) and Saudi nationals (92.2%), with 88.2% having one asthmatic child. Regarding awareness, exposure to air pollution (97.9%) and cigarettes (93.4%) were well-recognized factors. Pediatricians (50.5%) and family physicians (42.2%) were primary information sources. Concerning attitudes, most mothers disagreed with the harmful effects of inhalers (82.5%-92.7%) and advocated for avoiding smoking near asthmatic children (94.8%). Our study revealed that 94.5% of mothers of asthmatic children possessed a good level of knowledge about asthma in their children, while 5.5% demonstrated a low level of knowledge. Notably, mothers with good knowledge levels reported fewer emergency room visits (p=0.011) and hospitalizations (p=0.001). Predictors of good-level knowledge included higher education (adjusted odds ratio (AOR) =4.080, p=0.007) and absence of smoking relatives (AOR =0.222, p=0.005), while pet ownership was associated with lower knowledge (AOR =0.257, p=0.030).

Conclusion

Our study underscores the importance of maternal knowledge in pediatric asthma management. Good awareness levels were observed regarding key risk factors and appropriate attitudes toward medication use. Higher education and absence of smoking relatives were significant predictors of mothers’ knowledge of the disease.

## Introduction

Asthma is a chronic disorder of the airways characterized by obstruction and narrowing of breathing passage [1,2]. Asthma is a common condition in children, varying in severity from mild to severe symptoms. It often involves increased mucus production, bronchospasms, and airway wall edema, resulting in repeated episodes of airway obstruction [3]. The affected children may present with a variety of symptoms including wheezing, tightness of the chest, cough, and shortness of breath. Asthma has also been commonly associated with seasonal allergies and eczema [1,3].

Asthma in children as well as in adults is very common all over the world and the global estimates indicate that the number of patients affected with asthma may reach 300 million [3]. In children, the prevalence of asthma has been noted to increase and it is now presented as the most common chronic disease in children [4-6]. This increasing trend might be due to poor environmental conditions including a sedentary lifestyle, obesity, and exposure to different pathogens and pollutants [4,5].

Asthma diagnosis and management are challenging in the pediatric age group due to the wide spectrum of symptoms. A step-wise treatment protocol may help in controlling asthma and limiting the frequency of symptoms [7]. Managing asthma in both children and adults requires a nuanced approach, as each case may differ. Typically, strategies for controlling asthma in children involve both non-pharmacological and pharmacological methods. Non-pharmacological approaches focus on lifestyle changes and avoiding triggers. Caregivers are educated about common triggers like tobacco smoke, allergens in food, irritants in medications, and environmental pollutants. Training caregivers to alleviate asthma symptoms and regular clinical monitoring are also essential components of management [3,8].

Regarding medication-based treatment, various health agencies have compiled guidelines for managing asthma, including the Global Strategy for Asthma Management and Prevention, the British Thoracic Society/Scottish Intercollegiate Guideline Network, and the National Institute for Health and Care Excellence. These recommendations suggest incorporating an age-specific treatment approach in a stepwise manner by analyzing the severity of the disease and the level of control achieved by a specific therapy [9]. In addition, The Saudi Thoracic Society recently released updated guidelines called the Saudi Initiative for Asthma (SINA), offering detailed recommendations for diagnosing and managing asthma across different age groups [10]. Alruwaili et al. conducted a systematic review analyzing parental awareness of asthma globally, examining eight studies across various countries involving over 3700 parents. Their findings underscored a global lack of awareness among parents of asthmatic children, highlighting the necessity for interventions to enhance parental knowledge, attitudes, and practices regarding asthma management [11]. Mesbah et al. conducted a cross-sectional study in Egypt to examine mothers' behaviors, knowledge, attitudes, and practices regarding childhood bronchial asthma, involving over 130 participants. The study found that a majority (62.8%) of the mothers lacked sufficient knowledge about the subject [12]. Alharbi et al. presented findings from a cross-sectional survey evaluating local population awareness following an asthma campaign, collecting data from 475 participants and comparing results to a 2014 report. Participants scored an average asthma knowledge of 15.6 out of 25, with factors like age, personal asthma status, having asthmatic children, or knowing someone with asthma correlating with better awareness; the study noted significant differences compared to previous scores (70% vs. 63%) [13]. Another study examined the knowledge of parents or caregivers of asthmatic children regarding pediatric asthma. The study revealed a generally moderate level of knowledge about pediatric asthma among caregivers [14]. Addressing this issue requires heightened awareness among the general public especially mothers, who play a crucial role in early detection and management. By understanding asthma symptoms, causes, and prevention strategies, mothers can facilitate timely diagnosis and treatment. Therefore, assessing current maternal asthma awareness levels, determining the influencing factors, and examining their impact on childhood asthma control are imperative. The objectives of the study were to evaluate the knowledge and awareness levels among mothers of asthmatic children in Qassim region, Saudi Arabia, focus on the relationship between mothers' knowledge and awareness and disease control, and identify potential factors influencing their knowledge and awareness levels.

## Materials and methods

Study design and setting

A descriptive cross-sectional facility-based study was conducted in Qassim Region, Saudi Arabia, from January 2023 to June 2024, focusing on mothers with children aged one to 12 years diagnosed with asthma. The study took place in the primary healthcare facilities in the Qassim region. Thirty primary healthcare centers (PHCCs) out of a total of 155 PHCCs were randomly selected from five major cities: Buraydah, Unaizah, Bukayriyah, Al Rass, and Al Badaya in the Qassim region. We specifically targeted mothers who attended these facilities due to their accessibility and the geographical distribution of PHCCs, which cover most of the population clusters.

Sample size

The sample size of 422 participants was calculated using the Open EPi info program based on a 95% confidence interval, 5% margin of error, and a total of a selected population of Qassim region, Saudi Arabia which is 1,215,858 people, according to The General Authority for Statistics GASTAT [15]. The prevalence of asthma in the Qassim region of Saudi Arabia was 3.2% [16]. The estimated sample size was 384 and then was adjusted to 422 participants to compensate for the 10% non-response rate. Inclusion criteria involved mothers with asthmatic children residing in Qassim, able to read and write Arabic, and having access to social media. 

Data collection tool and procedures 

A structured self-administered questionnaire was designed after reviewing the literature review (asthma knowledge questionnaire and assessment of the level of asthma awareness among parents of children with asthma in Saudi Arabia) [17]. Data collection utilized an online self-administered questionnaire distributed via Google Forms.

The questionnaire was composed of three major sections: section one contained 11 questions about the personal characteristics including mothers’ age, occupation, residence, and education. Section 2 contained 26 close-ended questions for assessing the mothers’ knowledge and beliefs of asthma and its exaggeration factors. Section 3 contained 11 clinical questions assessing the control by using inhalers and the attitude of the mothers for treating their children during the attack.

Pilot study

A pilot study was conducted among 20 participants to ensure questionnaire clarity showing no modifications or changes in the questionnaire.

Data management and analysis

The data extracted from the Google Form was cleaned, entered into an Excel sheet, and analyzed using the computerized program IBM SPSS Statistics for Windows, Version 29 (Released 2023; IBM Corp., Armonk, New York, United States). The response for assessing the awareness of mothers regarding asthma is shown as poor and good knowledge used statistical analysis for the 26 knowledge questions. The knowledge questions’ answers were scored as follows: a score of “1” was given to the corrected answer (Yes), and a “0” score was given for both incorrect answers (No) or I don’t know. Mothers with a total knowledge score of 0-13 were categorized as “poor” knowledge and a score of 14-26 as “good” knowledge. 

Then the assessment of mothers’ knowledge level about asthma management is shown as poor, fair, or good categories using statistical analysis for the 11 knowledge questions. Mothers with a total knowledge score of 0-3 were categorized as “poor” knowledge, and a score of 4-6 as “fair” and of 7-11 as “good” knowledge. 

Categorical variables are reported as frequency (n), percentage, and continuous variables as mean ± standard deviation (SD) and range. Significant differences in the frequencies and percentages of categorical variables were analyzed by using the Chi-square test or Fisher's exact test. A p-value of <0.05 was considered statistically significant.

Ethical considerations

The research obtained institutional ethical clearance from the Qassim Region Ethics Committee, under approval number (607-44-14250), ensuring adherence to ethical guidelines. Confidentiality of all data was maintained, with its use strictly limited to research purposes.

## Results

Our study included 422 mothers of asthmatic children in the Qassim region of Saudi Arabia (Table 1). Most participants were aged between 35 and 44 years (n=214, 50.7%), followed by those aged≤34 years (n=167, 39.6%). Most were Saudi nationals (n=389, 92.2%) and currently married (n=382,90.5%). About 88.2% of mothers reported having one child diagnosed with asthma (n=372), while 11.8% had two or more asthmatic children (n=50). Most asthmatic children were five years and older (n=307, 72.7%). Additionally, 58.1% of mothers reported having at least one family member diagnosed with asthma (n=245). Furthermore, 82.5% had a university-level education (n=348), and 93.1% had a monthly income of less than 5,000 Riyal (n=393). Moreover, 9.7% of households reported current smoking by a family member (n=41), and 13.7% reported having pets at home (n=58), with cats being the most common (n=40, 9.5%).

**Table 1:** Sociodemographic parameters of mothers of asthmatic children (n=422) (N) Frequency, (%) Percentages

Characteristics		N (%)
Age (years)	≤34	167 (39.6)
	35-44	214 (50.7)
	45-54	40 (9.5)
	≥55	1 (0.2)
Nationality	Saudi	389 (92.2)
	Non-Saudi	33 (7.8)
Marital status	Currently Married	382 (90.5)
	Divorced/Widowed	40 (9.5)
No. of children with asthma	1 child	372 (88.2)
	≥ 2 children	50 (11.8)
Age of the asthmatic child	< 5 years	115 (27.3)
	≥ 5 years	307 (72.7)
Any family member diagnosed with asthma	Yes	245 (58.1)
Mother education level	High school/ Less	57 (13.4)
	University	348 (82.5)
	Postgrad	17 (4.0)
Monthly income	< 5000 Riyals	393 (93.1)
	5000-10000 Riyals	7 (1.7)
	10000-15000 Riyals	15 (3.6)
	> 15000 Riyals	7 (1.7)
Anyone in family smoker currently	Yes	41 (9.7)
House type	Apartment	166 (39.3)
	Villa	254 (60.2)
	Farm	2 (0.5)
Any pet animal at home	Yes	58 (13.7)
Which animal	Cat	40 (9.5)
	Birds	17 (4.0)
	Rabbit	1 (0.2)

Table 2 assesses mothers' knowledge and awareness regarding asthma risk factors and other related features. Among identified risk factors, the highest level of awareness was observed for exposure to dust or air pollution (n=413, 97.9%), followed closely by exposure to cigarettes, either directly or indirectly (n=394, 93.4%), and exposure to perfume (n=387, 91.7%). Chronic diseases with acute exacerbations (n=360, 85.3%) and the importance of regular treatment to prevent asthma attacks (n=343, 81.3%) were also well-recognized factors. Factors like influenza as a cause of asthma (n=309, 73.2%) and using inhalers during the flu (n=306, 72.5%) were widely recognized as factors by the mothers. Climate change (n=295, 69.9%) and contact with pets (n=241, 57.1%) were the least recognized factors. Regarding other features, awareness was notably high for recognizing asthma symptoms like dyspnea (n=339, 80.3%) and understanding severe asthmatic attacks' potential consequences, including ICU admission or death (n=330, 78.2%). However, awareness was relatively lower for concepts like medicines requiring daily use for effectiveness (n=320, 75.8%) and the inheritance of asthma (n=314, 74.4%).

**Table 2:** Assessment of knowledge and awareness of mothers about asthma, risk factors, and other features (n=422) (N) Frequency, (%) Percentages PHCC: Primary healthcare center

Knowledge and Awareness Questions		N (%)
Asthma is a chronic disease with acute exacerbations on exposure to allergens	Uncertain/No	62 (14.7)
	Yes	360 (85.3)
Is asthma an inherited disease?	Uncertain/No	108 (25.6)
	Yes	314 (74.4)
The causes and irritants of asthma air pollution or dust	Uncertain/No	9(2.1)
	Yes	413 (97.9)
Contact with pets	Uncertain/No	181 (42.9)
	Yes	241 (57.1)
Climate change (from cold to hot)?	Uncertain/No	127 (30.1)
	Yes	295 (69.9)
Influenza Infection	Uncertain/No	113(26.8)
	Yes	309(73.2)
Exposure to smoke (cigarettes) directly or indirectly	Uncertain/No	28(6.6)
	Yes	394 (93.4)
Exposure to perfumes, incense or paint fumes could lead to acute asthma attacks	Uncertain/No	35(8.3)
	Yes	387 (91.7)
Symptoms of asthma include	Dyspnea	339 (91.7)
	Sore Throat	1 (0.2)
It is possible to avoid an asthma attack if the treatment is taken regularly, even if the symptoms of the disease are not apparent between attacks	Uncertain/No	79 (18.7)
	Yes	343 (81.3)
When a child has the flu, the inhaler should be used even if the symptoms of the disease are not apparent such as coughing or wheezing	Uncertain/No	116 (27.5)
	Yes	306 (72.5)
Children with asthma may have severe attacks that require hospitalization, admission to the intensive care unit, or lead to death	Uncertain/No	92 (21.8)
	Yes	330 (78.2)
Some asthma medications do not work unless they are used daily	Uncertain/No	102 (24.2)
	Yes	320 (75.8)
Did you get instructions either in hospital or PHCC?	No	28 (6.6)
	Yes	394 (93.4)

**Figure 1:**
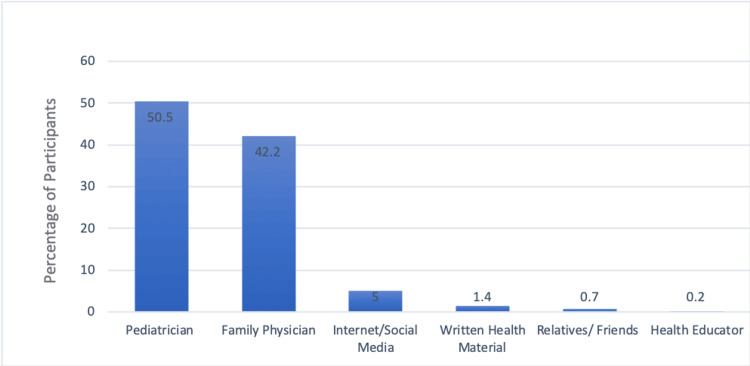
Source of information of mothers about knowledge and awareness of asthma

Figure 1 shows the primary sources of information for mothers regarding knowledge and awareness of asthma. According to the data, the most common source of information is pediatricians, accounting for 50.5% (n=213). Family physicians also play a significant role, with 42.2% (n=178) of mothers obtaining information from them. A smaller proportion, 5%, rely on the Internet and social media for information about asthma. Written health materials contribute to the knowledge of only 1.4% (n=6) of mothers, while information from relatives or friends is minimal, at 0.7% (n=3). Health educators have the least impact, with only 0.2% (n=1) of mothers citing them as a source of information.

Table 3 shows the attitudes and beliefs of mothers towards asthma management for their children. A significant portion of mothers was unsure or not aware of the notion that inhaler use could lead to addiction or dependence (82.5%) with an answer no or uncertain n=348) and that inhalers could harm the heart (92.7% no or uncertain n=391). Similarly, a majority were uncertain or not aware that using sprays for prolonged periods was harmful (89.6% no or uncertain n=378) and that medication should be stopped after the cough subsides during an asthma attack (81.8% no or uncertain n=345). Additionally, a considerable proportion was unsure or not aware of the idea that children should only use medications during an attack (75.6% no or uncertain n=319). Furthermore, many were uncertain or not aware of the use of nebulizers without a spacer (76.3% no or didn't know n=322), and the necessity of taking a child to the emergency room (ER) even for mild symptoms (71.3% no or uncertain, n=301). Regarding alternative treatments, a significant portion did not use herbal medicines alongside asthma medications (82.2% no, n=347).

**Table 3:** Attitudes and beliefs of mothers towards asthma of children (n=422) (N) Frequency, (%) Percentages

Attitudes and Beliefs Questions		N(%)
Inhaler use may lead to addiction or dependence	Uncertain/No	348 (82.5)
	Yes	74 (17.5)
The inhaler may affect or harm the heart	Uncertain/No	391 (92.7)
	Yes	31 (7.3)
Using spray for children for long periods is not a good thing	Uncertain/No	378(89.6)
	Yes	44 (10.4)
The use of inhalers and medicines should be discontinued after the cough stops in a child having an asthma attack	Uncertain/No	345 (81.8)
	Yes	77 (18.2)
Children with asthma should only use medications to treat an attack when they notice signs (coughing, congestion, or wheezing)	Uncertain/No	319 (75.6)
	Yes	103 (24.4)
It is best to use the nebulizer directly without a spacer, as this leads to the drug directly entering the lungs	Uncertain/No	322(76.3)
	Yes	100(23.7)
When a child has asthma attack, it is best to take him to the emergency room even if the symptoms are mild	Uncertain/No	301(71.3)
	Yes	121 (28.7)
I think and belive herbal medicines are helpful in asthma treatment?	Uncertain/No	347(82.2)
	Yes	75 (17.8)

Table 4 shows various aspects of asthma control in children as perceived by their mothers. Most mothers believed that asthmatic children should not participate in strenuous sports activities (64.9%, n=274). Additionally, most mothers recognized the importance of avoiding smoking around asthmatic children (94.8%, n=400). A significant proportion reported their child visiting the ER due to asthma in the past year (72.5% yes, n=306), with a notable frequency of ER visits, including one-time (28.0%, n=118), two times (23.7%, n=100), and three or more times (21.1%, n=89). Hospitalization due to asthma was reported by 30.1% (n=127) of mothers, with varying frequencies (one time: 23.5%, n=99; two or more times: 6.6%, n=28). Regarding medication, most mothers reported using both controller and reliever therapy (55.7%, n=235). Instructions on inhaler use were well understood by the majority, including exhaling fully before use (74.4%, n=314), closing lips tightly around the mouthpiece (89.8%, n=379), breathing deeply through the mouthpiece (92.9%, n=392), holding breath for at least 10 seconds after inhalation (57.3%, n=242), and exhaling slowly after use (59.2%, n=250).

**Table 4:** Different features showing the control of asthma of children (n=422) (N) Frequency, (%) Percentages

Question about the control of asthma		N (%)
Children with asthma should be prevented from participating in sports activities that require them to run a lot	Uncertain/No	148 (35.1)
	Yes	274 (64.9)
It is best not to smoke and not to allow anyone to smoke near an asthmatic child	Uncertain/No	22(5.2)
	Yes	400 (94.8)
Has your child visited the emergency room because of asthma in the past year	No	116 (27.5)
	Yes	306 (72.5)
If yes, how many times has your child visited the ER for asthma in the past year?	One Time	118 (28.0)
	Two Times	89 (21.1)
	≥ Three Times	89 (21.1)
Has your child been hospitalized because of asthma in the past year?	No	295 (69.9)
	Yes	127 (30.1)
If yes, how often was your child hospitalized because of asthma in the last year?	One Time	99 (23.5)
	≥ Two Times	28 (6.6)
What type of asthma medication do you use for your child?	Controller therapy	84 (19.9)
	Reliever therapy	95 (22.5)
	Both	235 (55.7)
	Others	8 (1.9)
Exhale fully before use	Uncertain/No	108 (25.6)
	Yes	314 (74.4)
Close the lips tightly around the mouth of the inhaler	Uncertain/No	43 (10.1)
	Yes	379 (89.8)
Breath in deeply through the Mouthpiece	Uncertain/No	30 (7.1)
	Yes	392 (92.9)
Hold your breath for at least 10 s after breathing in	Uncertain/No	180 (42.7)
	Yes	242 (57.3)
Breath out very slowly after use	Uncertain/No	172 (40.8)
	Yes	250 (59.2)

**Figure 2:**
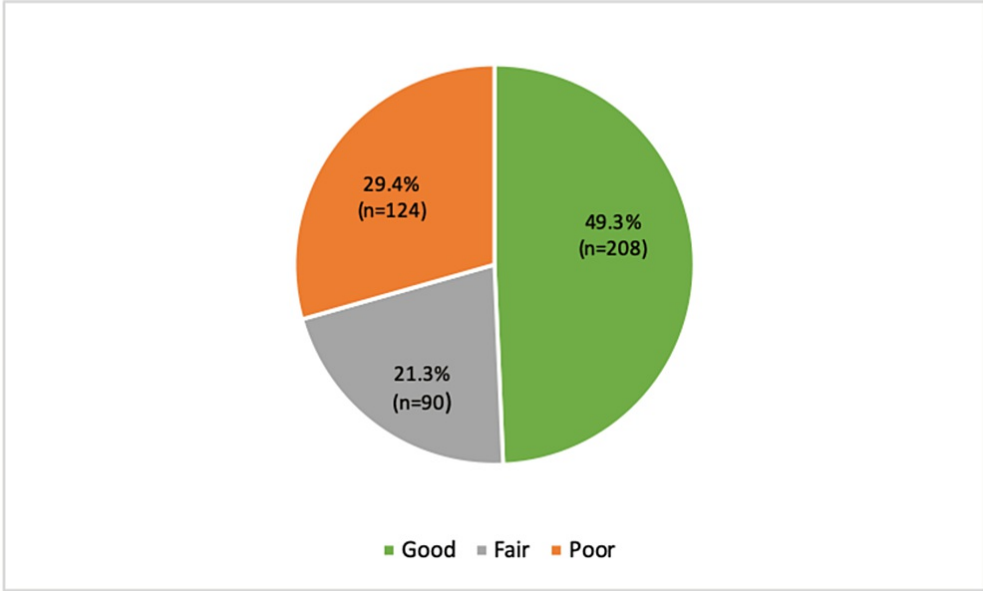
Knowledge and awareness level of mothers about asthma of their child (n) Frequency, (%) Percentage

Figure 2 shows the knowledge level of mothers about asthma management for their children. The distribution indicates that almost half of the mothers possessed good knowledge (49.3%, n=208), followed by a fair level (21.3%, n=90) and a poor level of knowledge (29.4%, n=124).

Table 5 shows the association between the knowledge level of mothers about asthma and its impact on asthma control. Mothers with good knowledge levels were significantly more likely to report that their child had not visited the ER due to asthma in the past year than those with poor knowledge (99.1% vs. 0.9%, p=0.011). Similarly, children of mothers with good knowledge levels were less likely to have visited the ER multiple times (>4 times) in the past year. Regarding hospitalization due to asthma, although not statistically significant, there was a trend towards fewer hospitalizations among children of mothers with good knowledge levels (95.9% vs. 4.1%, no hospitalization, p=0.057). However, among those hospitalized, children of mothers with good knowledge were less likely to be hospitalized multiple times (77.8% for >2 times) (p<0.001). Asthma control status showed no significant association with the maternal knowledge level (p=0.116).

**Table 5:** Association between the knowledge level of mothers about asthma and its impact on asthma control ^a^Chi-Square Test, ^b^Fisher's Exact Test

		Knowledge /Awareness of mothers about asthma		p-value
		Poor knowledge	Good knowledge	
		N (%)	N (%)	
Total		23(5.5)	399 (94.5)	
Has your child visited the	No	1 (0.9)	115 (99.1)	0.011^a^
emergency room because of asthma in the past year?	Yes	22 (7.2)	284 (92.8)	
If yes, how many times has your child visited the ER for asthma in the past year	1 Time	5 (4.2)	113 (95.8)	0.006 ^b^
	2 Times	5 (5.0)	95 (95.0)	
	3 Times	3 (6.7)	42 (93.3)	
	4 Times	4 (14.8)	23 (85.2)	
	>4 Times	5 (29.4)	12(70.6)	
Has your child been hospitalized because of asthma in the past year?	No	12 (4.0)	283(95.9)	0.057^ a^
	Yes	11 (8.7)	116(91.3)	
If yes, how often was your child hospitalized because of asthma in the last year	1 Time	4 (4.0)	95 (96.0)	<0.001^ b^
	2 Times	6 (22.2)	21 (77.8)	
	3 times	1(100.0)	0 (0)	
Based on above features, asthma controlled or not controlled	Controlled	5 (4.2)	113 (95.8)	0.116^a^
	Uncontrolled	17 (9.0)	172 (91.0)	

Table 6 shows the adjusted predictors of good-level knowledge about asthma among mothers as determined by multivariate analysis. The results reveal that higher education significantly predicts good-level knowledge, with an adjusted odds ratio (AOR) of 4.080 and a p-value of 0.007. Conversely, having a smoking father or relative is associated with lower levels of good-level knowledge, with an AOR of 0.222 and a p-value of 0.005. Additionally, the presence of pets at home is maybe linked to lower levels of knowledge, with an AOR of 0.257 and a p-value of 0.030.

**Table 6:** Adjusted predictor of good-level knowledge about asthma (multivariate analysis)

	B	p-value	Adjusted odds ratio (AOR)	95% CI	
				Lower	Upper
Age of the mother	.023	.657	1.024	.924	1.134
Nationality (non-Saudi)	-.071	.920	.931	.232	3.735
Marital status	-.188	.767	.829	.240	2.866
No. of children with asthma	.386	.601	1.471	.346	6.254
Age of the asthmatic child	-.106	.337	.899	.724	1.117
Family member with asthma	.916	.059	2.499	.965	6.474
Higher education	1.406	.007	4.080	1.480	11.242
Higher family income	-.171	.713	.843	.338	2.100
Family member is a smoker	-1.507	.005	.222	.077	.638
Type of house	.588	.336	1.800	.544	5.958
Pet at home	-1.360	.030	.257	.075	.875
Constant	-4.626	.174	.010		

## Discussion

Our study provides valuable insights into the profile of mothers of asthmatic children in the Qassim region of Saudi Arabia. The predominance of mothers aged between 35 and 44 years reflects the typical age range of parents with school-aged children. The high proportion of Saudi nationals and currently married participants is consistent with Saudi society's cultural norms and family structures.

Furthermore, the majority of participants had a university-level education and a monthly income of less than 5000 Saudi Riyal, indicating that education and socioeconomic status may influence health-seeking behaviors and access to healthcare services. Mou et al. showed that socio-demographic factors significantly influenced health-seeking behaviors in the Bangladeshi population [18]. Notably, our findings indicate almost half of the mothers have a good knowledge level (49.3%) regarding various asthma risk factors. Similarly, a study by Fasola et al. showed that the majority of mothers have good knowledge (67%) regarding asthma [19]. Factors such as exposure to air pollution or dust, exposure to cigarettes, and the importance of regular treatment to prevent asthma attacks were well-recognized. Moreover, Tiotiu et al. showed that traffic-related air pollution, nitrogen dioxide, and second-hand smoking exposures represent significant risk factors for asthma development in children [20]. However, there were gaps in awareness, particularly regarding climate change and contact with pets as asthma triggers. Similarly, a systematic review of 32 studies by Apelberg et al. suggested that pet exposure was associated with a slight increase in the risk of asthma and wheezing in older children [21]. These findings suggest the need for targeted educational interventions to improve awareness of less recognized risk factors among mothers of asthmatic children.

Pediatricians, accounting for 50.5%, emerged as the primary source of information for mothers regarding asthma, followed by family physicians at 42.2%. This underscores the pivotal role of healthcare providers in educating and empowering mothers to manage their children's asthma crises effectively. The limited reliance on the Internet and social media highlights the importance of credible sources of information in shaping mothers' knowledge and attitudes toward asthma management. Similarly, AlOtaibi et al. showed various sources of information of participants about asthma such as pediatricians, family physicians, written materials, and the Internet [14].

Moreover, the majority of mothers expressed uncertainty or disagreed with misconceptions surrounding asthma management, such as concerns about inhaler addiction or dependence and the perceived harm of inhalers to the heart. Similarly, Zaraket et al. showed various concerns among parents such as inhalers being addictive, worried about inhalers’ side effects, and worried about using inhaled corticosteroids [22]. These findings are encouraging as they indicate a generally positive attitude toward evidence-based asthma treatments among mothers in the Qassim region. However, there remains room for improvement in addressing misconceptions and enhancing understanding of optimal asthma management practices.

Moreover, our study revealed a significant proportion of asthmatic children visiting the ER (72.5%) and being hospitalized due to asthma exacerbations. Similarly, Alfurayh et al. showed that the frequency of hospital admissions and pediatric ER visits due to asthma exacerbation is increasing [23]. While the majority of mothers reported using both controller and reliever therapy for their children, the frequency of ER visits and hospitalizations underscores the need for improved asthma control and management strategies. Furthermore, the association between the maternal knowledge level and ER visits highlights the potential role of education in preventing asthma exacerbations and reducing healthcare utilization. Similarly, Alsayed et al. and Limaye et al. showed that the extent of asthma control was significantly associated with caregivers' knowledge and practices for children with asthma [24,25].

Multivariate analysis in our study identified several predictors of good-level knowledge about asthma among mothers, including higher education and the absence of smoking family members. These findings are consistent with previous studies highlighting the importance of education and household environment in shaping health literacy and awareness of chronic diseases such as asthma. Additionally, the presence of pets at home was associated with lower levels of knowledge, suggesting the need for targeted education and interventions for households with pets to improve asthma management practices. Moreover, Gallop et al. showed various predictors of higher asthma knowledge including higher household income, fewer children, and greater negative impacts from asthma. Self-efficacy was predicted by fewer barriers to a child's healthcare [26].

Our study supports existing research emphasizing maternal knowledge's significance in asthma management. Healthcare providers are crucial information sources, requiring tailored interventions. Additionally, our findings offer new insights into demographic characteristics and healthcare utilization among mothers in Saudi Arabia's Qassim region.

Several limitations should be acknowledged when interpreting the findings of our study. Firstly, the cross-sectional design limits our ability to establish causality between maternal knowledge and asthma outcomes. Additionally, the reliance on self-reported data may introduce recall bias and social desirability bias, potentially influencing the accuracy of responses. Furthermore, the generalizability of our findings may be limited to the Qassim region of Saudi Arabia and may not reflect the experiences of mothers in other regions or countries.

Our study highlights the need for targeted educational interventions to improve maternal knowledge of asthma management in the Qassim region. Future research should explore the effectiveness of such interventions in improving asthma control and reducing healthcare service utilization among children. Additionally, investigating cultural and contextual factors influencing asthma management is essential for tailored intervention development.

## Conclusions

Our study provides valuable insights into the knowledge of mothers of asthmatic children in the Qassim region of Saudi Arabia. Even though maternal awareness of asthma risk factors and optimal management practices is nearly half good, there are opportunities for targeted education and interventions to address knowledge gaps and improve asthma control outcomes.

Healthcare providers play a crucial role in empowering mothers with accurate information and promoting evidence-based asthma management strategies. Future research should focus on longitudinal studies to assess the impact of educational interventions on asthma outcomes.
